# A Comparison of the Efficacy of 5 mg Olanzapine and Aprepitant in the Prevention of Multiple-Day Cisplatin Chemotherapy-Induced Nausea and Vomiting

**DOI:** 10.1155/2022/5954379

**Published:** 2022-09-07

**Authors:** Guang Liu, Yilan Jin, Ying Jiang, Juan Zhao, Caihong Jiang, Zewei Zhang, Lanzhen Zhao, Hui Li, Feng Chen, Jing Wang, Hui Fan, Zhenhao Li, Yongqiang Jia, Gaowa Jin, Quanfu Li

**Affiliations:** ^1^Department of Medical Oncology, Ordos Central Hospital, Ordos 017000, China; ^2^Department of Radiotherapy, Tong Liao City Hospital, Tong Liao 028000, China; ^3^Department of Radiation Oncology, Sun Yat-sen University Cancer Center, State Key Laboratory of Oncology, South China, Collaborative Innovation Center for Cancer Medicine, Guangzhou 510000, Guangdong, China; ^4^School of Public Health and Management, Wenzhou Medical University, Wenzhou 325000, Zhejiang, China

## Abstract

**Objective:**

The significance of this article is to talk about aprepitant and olanzapine 5 mg, compare them, and deeply explore the safety or effectiveness during the whole process of multiple-day cisplatin chemotherapy-induced vomiting and nausea.

**Methods:**

This trial was randomized and prospective. It is needed to receive cisplatin chemotherapy (25 mg/m2/d) for three days. Its patients would need to choose to use 5 mg olanzapine or aprepitant for this treatment, combined with 5-HT3 receptor antagonist plus dexamethasone. The primary endpoints were the total protection (TP) during the acute phase (AP) (0–24 hours), delayed phase (DP) (25–120 hours), and overall phase (OP) (0–120 h) between the two groups. The secondary endpoints were the complete response (CR) and total control (TC) during the three phases. The first time of the whole process is particularly important and needs to be observed vigorously. However, the time of the patient's first vomiting symptom is also compared accurately by using the Kaplan–Meier curve. The functional life index vomiting (FLIE) was used to calculate and carefully evaluate the serious impact of nausea and vomiting (CINV) induced by the whole chemotherapy process on the quality of life. About olanzapine, its related symptoms and other side effects and aprepitant were also recorded.

**Results:**

(1) The primary endpoint TP rates of the olanzapine and aprepitant groups were similar; for the AP, they were 94.23% (98/104) vs. 95.45% (98/106) *P*=0.61(*P*=0.61); for the DP, they were 54.81% (57/104) vs. 54.72% (58/106) (*P*=0.99), and for the OP, the values were 53.79% (58/105) and 55.31% (56/104), respectively (*P*=0.99). The secondary endpoints, the TC rates, and CR rates were also comparable in the three phases (*P* > 0.05). (2) After research and display, the results showed that there was no significant difference between the two groups when they were used for the first time of vomiting and the FLIE index (*P* > 0.05). (3) The main olanzapine-related adverse event was drowsiness, while that of aprepitant was constipation.

**Conclusion:**

The efficacy of 5 mg olanzapine was similar to that of aprepitant, and it also showed an advantageous economic potency ratio in preventing CINV induced by multiple-day cisplatin chemotherapy with increased sedation side effects.

## 1. Introduction

After many clinical types of research and observations involving cisplatin multiple-day chemotherapy, the results show that when aprepitant, 5-HT3 receptor antagonist (5-HT3RA) and dexamethasone (DXM) are used together, and the complete response (CR) is much higher than that when 5-HT3RA and DXM are used alone [1, 2]. However, the condition of nausea and vomiting in patients is still a problem that needs to be discussed and studied [3]. Olanzapine, an atypical antipsychotic drug, can inhibit well-established symptoms of nausea and vomiting. It is an important pathway of neurotransmitters closely related to olanzapine, including dopamine and *α*-adrenergic receptors, histamine, and serotonin receptors, and its role in chemotherapy-induced nausea and vomiting (CINV) has attracted increasing attention [4]. Previous studies have confirmed that the efficacy of 10 mg olanzapine is similar to that of aprepitant in the prevention of CINV, and olanzapine will have stronger and better inhibition of nausea symptoms in the delayed phase (DP) and overall phase (OP) [5]. When the drug dose of olanzapine is 10 mg, the patient will have drowsiness, which is also the main side effect. When entering the second stage of in-depth research, the activities of olanzapine 5 mg and olanzapine 10 mg are equal, which will be safer for drowsiness symptoms and in good condition[6]. Patients receiving multiple-day cisplatin chemotherapy are at risk of both acute and delayed nausea and vomiting each day, as acute and delayed emesis may overlap after the initial day of chemotherapy until the last day of chemotherapy [7, 8]. Therefore, we conducted a more prospective comparison and in-depth discussion and then studied carefully 5 mg olanzapine or aprepitant was used together with 5-HT3RA and DXM to prevent the therapeutic effect of CINV induced by multiple-day cisplatin chemotherapy.

## 2. Methods

### 2.1. Patients

A randomized clinical trial (ChiCTR2000036826) was conducted by the Department of Oncology at Ordos Central Hospital to the effect and availability of CINV in patients who received cisplatin chemotherapy for three days after using olanzapine with 5 mg or aprepitant with 5-HT3RA and DXM. This study was also reviewed by local institutions and approved by the review committee. All patients receiving treatment need to provide written informed consent in advance.

### 2.2. Endpoints

The total protection (TP) rate was chosen as the primary endpoint. Total protection was defined as no vomiting symptoms during OP. On the 100 mm nausea research score table, the maximum value of the nausea score is 25 mm. The secondary endpoints were the complete response (CR) and total control (TC). CR refers to no vomiting symptoms and no use of rescue drugs. TC refers to no vomiting and no timely use of rescue drugs. In the 100 mm, the nausea scale score is less than 5 mm. The occurrence of adverse events (AE) was graded according to Common Terminology Criteria v.4.0.

### 2.3. Randomization

Once it was necessary to accurately and carefully verify whether the patient meets the standards and actively confirm whether the patient is qualified, the patient was randomized using a random number table.

### 2.4. Eligibility Criteria

#### 2.4.1. Inclusion Criteria

The specific criteria: (1) patients over the age of 18, with histological confirmation, receiving three-day cisplatin-based chemotherapy (25 mg/m^2^/d); (2) patients with a Karnofsky Performance Scale result ≥70; (3) a comprehensive examination is required before patients start chemotherapy. For all patients whose liver and kidney functions, blood routine, and ECG are not abnormal, including those whose white blood cell count is >3.5 × 10^9^/L, the absolute number of cells with neutrophils is more than 1.5 × 10^9^/L and platelet count >× 10^9^/L, the value of alkaline phosphatase <2.5 is its upper normal limit (ULN), the value of alanine aminotransferase <2.5 ULN, the value of bilirubin <1.5 ULN, and the value of creatinine <1.5 ULN; (4) patients with no symptoms of nausea and vomiting, who had not been treated with aprepitant or olanzapine in the week before enrollment; (5) for those patients without chemotherapy contraindications; (6) those patients who can fully understand and clearly describe the reported results.

#### 2.4.2. Exclusion Criteria

The exclusion criteria are (1) patients who cannot take oral drugs by themselves; (2) sick patients who had vomited 24 hours before the chemotherapy; (3) those who need abdominal or pelvic radiotherapy; (4) patients who need to be treated with some cortical sterols; (5) pregnant and lactating women, or women with reproductive potential, and men who are eager to have children; (6) patients with symptomatic brain metastases; (7) patients with partial/complete bowel obstruction; (8) patients who were taking quinolone antibiotics; (9) patients who were users of illegal drugs or alcohol; (10) patients with allergic symptoms or history of allergy to research drugs or such compounds; (11) patients with gastrointestinal malignancies.

### 2.5. Treatment Methods

In this article, the antiemetic agents used can refer to Table 1. The patients in the olanzapine antiemetic regimen group received the following: olanzapine 5 mg PO days 1–4, tropisetron 5 mg IV days 1–3 (Beijing Shuanglu Pharmaceutical Co. Ltd., China), and DXM 10 mg IV days 1–3. And the table need correct the location of day 4 of olanzapine 5 mg. Patients in the aprepitant regimen group can be treated with aprepitant 125 mg PO for the first day, 80 mg PO for the second to third days (EMEND, MSD Sharp and Dohme, Haar, Germany), tropisetron 5 mg IV for the first to third days, and DXM 5 mg IV for the first to third days. The aprepitant group had a half dosage of DXM, besides the tropisetron hydrochloride and aprepitant, since the function of CYP3A4 in DXM pharmacokinetics can be exhibited by aprepitant [9].

### 2.6. Follow-Up

Infusion was done during chemotherapy (from 0 hours) until the fifth day. Patients recorded and self-reported the times and dates of vomiting or retching episodes and the use of rescue treatments, from the time of chemotherapy infusion (0 h) until day 5. In the morning of the 2nd–5th day, it is necessary to contact and check with the patients, to accurately ensure that the patients meet the classification standards and indicators of nausea grade. The functional life index nausea and vomiting (FLIE) questionnaire will carry out early self-description and management on day 5, so that the final patient self-recording report will be completed [10]. What deserves more attention and research is that the file is a fact-tested questionnaire specially designated for nausea and vomiting, which contains 9 important questions in the field of nausea (important items) and 9 important questions in the field of vomiting (important items). The display of “CINV has no impact on daily life” in the 7 subscale shows that the average score is >6 (total value >108) [11, 12].

All patients undergoing treatment need to undergo and receive a comprehensive examination on days 6 after the treatment process and then visit again and record on days 19–21. Any adverse events related to aprepitant and olanzapine will be recorded one by one.

### 2.7. Statistical Analysis

SPSS25.0 software was used to analyze and process the data. Chi-square test was used to compare the percentage value of patients in the two groups who achieved TP, CR, and TC or experienced related AEs. The time and the date of the first vomiting and Kaplan–Meier curve were drawn at the same time. It was considered that *P* < 0.05, which also fully shows that it is statistically significant.

## 3. Results

### 3.1. Study Patients

The time and date of this prospective randomized controlled study are from March 2019 to December 2020. This study was conducted in the Department of oncology, Ordos Central Hospital, Inner Mongolia, China. A total of 222 patients will be randomized into two groups. Twelve patients were later withdrawn because of poor compliance with the doctor's advice, refusing to accept the aprepitant or stopping the chemotherapy due to a change in their illness. Thus, 210 follow-up records were eventually included in the analysis, there were 104 patients in the olanzapine group and 106 patients in the aprepitant group. All patients will receive cisplatin-based 3-day dual regimen and cooperate with one of the following drugs for chemotherapy: gemcitabine, docetaxel, etoposide, pemetrexed, paclitaxel, capecitabine, or irinotecan, sometimes with the addition of bevacizumab.  Table 2 shows that the baseline characteristics of the two groups are highly comparable.

### 3.2. Efficacy

**Figure 1:**
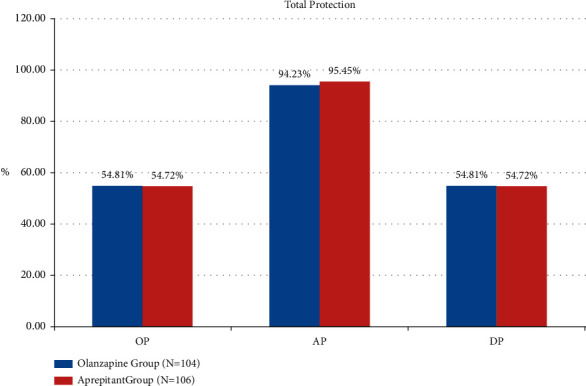
Comparison of total protection between two groups.

**Figure 2:**
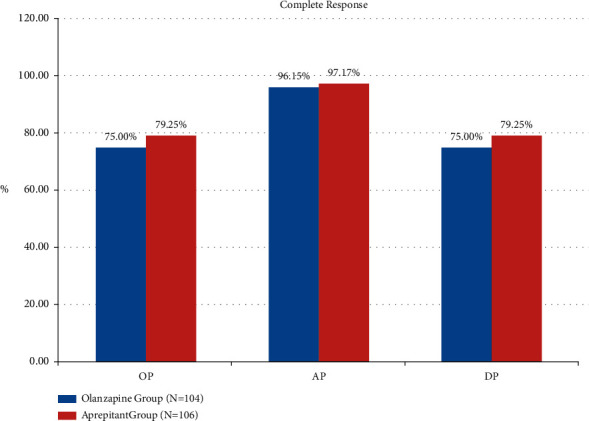
Comparison of complete response between two groups.

**Figure 3:**
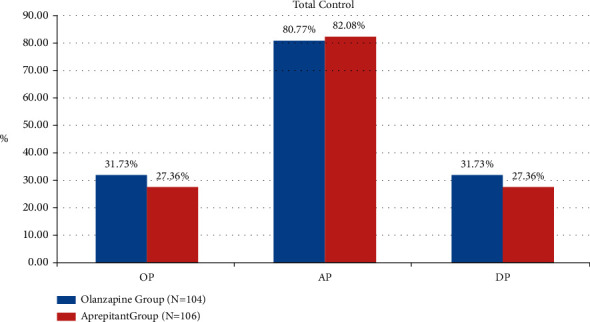
Comparison of total control between two groups.

The TP rates in olanzapine group and aprepitant group were 94.23% (98/104) vs. 95.45% (98/106) (*P*=0.61), 54.81% (57/104) vs. 54.72% (58/106) (*P*=0.99), and 54.81% (57/104) vs. 54.72% (58/106) (*P*=0.99) in the acute phase, delayed phase, and overall phase, respectively. As can be seen from Figure 1, among DP, the TP rate of olanzapine group is better than that of the aprepitant group. As can be seen from Figure 2, the secondary endpoint CR rates in the olanzapine group and aprepitant group were 96.15% vs. 97.17% (*P*=0.98), 75.00% vs. 79.25% (*P*=0.46), and 75.00% vs. 79.25% (*P*=0.46) in the three phases, respectively, as shown in Figure 2. It can be seen from Figure 3 that the TC rates in the olanzapine group and aprepitant group were 80.77% vs. 82.08% (*P*=0.81), 31.73% vs. 27.36% (*P*=0.49), and 31.73% vs. 27.36% (*P*=0.49) in the three phases, respectively. The results of the two groups in the indicators described above can show that there is no statistical difference between the two groups.

### 3.3. The Comparison of the FLIE

In terms of the FLIE, for CINV, it has no impact on patients' daily life. Such reports are also explained and displayed by 63.46% (66/104) of the olanzapine group and 66.04% (70/106). The value of the aprepitant group is *P*=0.036. The results of the FLIE can be seen in detail in Table 3.

### 3.4. The Comparison of Time to First Vomiting

**Figure 4:**
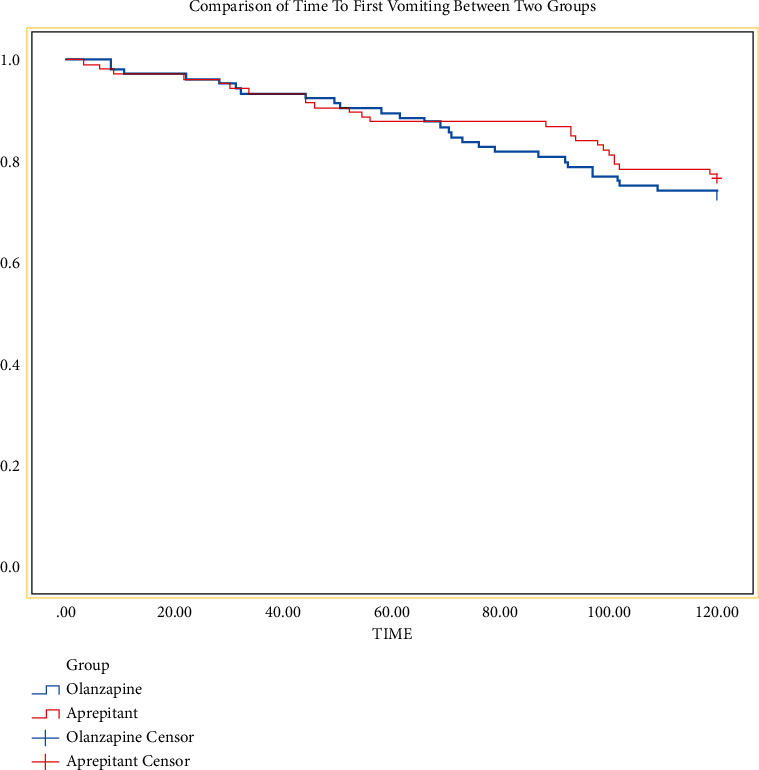
Comparison of time to first vomiting between two groups.

The Kaplan–Meier curves (seen in Figure 4) show that the first vomiting occurred aprepitant group will be later than olanzapine group, but there was no statistical difference (*P*=0.57).

The most common AE Olanzapine's team was somnolence, while that in the aprepitant group was constipation. These two AEs were observed in 88.46% (92/104) and 52.88% (55/104) of patients in the olanzapine regimen group and 50.8% (66/106) and 62.26% (66/106) of patients in the aprepitant regimen group, respectively (*P*=0.00 and *P*=0.02). There was no statistical significance in AEs such as hiccupping, fatigue, dizziness, headache, loss of appetite, and abdominal distension (*P* > 0.05). There were no adverse events in grade 3 or 4 of the study due to a side effect of severe drowsiness.

## 4. Discussion

Rudolph et al. conducted a phase III randomized controlled clinical trial to compare 10 mg olanzapine, and this is actually a combination of 5-HTRA3A and DXM was the need to prevent CINV in advance that led to it, by cyclophosphamide + adriamycin or one-day cisplatin chemotherapy in 241 patients. In the olanzapine and aprepitant groups, the percentage of acute or delayed CR was 97% vs. 87% (*P* > 0.05) and 77% vs. 73% (*P* > 0.05), respectively, in the absence of nausea, the proportion was 86% and 86% (*P* > 0.05) and 68% and 37% (*P* < 0.01), respectively [5]. Both of them had similar efficacy in relieving CINV induced by highly emetogenic chemotherapy (HEC), and olanzapine achieved a higher nausea control rate during DP [5]. Multiple studies and systematic reviews have confirmed that olanzapine is as effective as neurokinin-1 receptor antagonism in the prevention of CINV induced by moderate emetic drugs and HEC [13–15]. Due to the sedative effect of olanzapine, up to 73% of patients receiving 10 mg olanzapine will experience drowsiness [16]. To reduce AEs, further research and exploration are needed to reduce the dose of olanzapine. A phase II randomized double-blind study conducted by Takako et al. showed that the incidence of drowsiness was very low with 5 mg olanzapine compared with 10 mg olanzapine [6].

To further evaluate the efficacy difference between 5 mg olanzapine and aprepitant in the prevention of CINV induced by multiple-day cisplatin chemotherapy, the Department of Oncology at Ordos Central Hospital conducted the first clinical study of 5 mg olanzapine or aprepitant, in combination with 5-HT3RA and DMX. In this study and discussion, the primary endpoint TP rate and secondary endpoint CR rate or TC rate were targeted. During the AP, DP, and OP phase, either the TP rate or the CR and TC rate of the olanzapine group was very similar to that of the aprepitant group. Rather than the CR rate used in Rudolph et al.'s phase III randomized controlled trial, this study used the TP rate, which focuses more on nausea assessment, as the main endpoint [5]. The TP rates of the olanzapine group were in line with those of the aprepitant group during the AP (94.23% vs. 95.45%, *P*=0.61) and the DP (54.81% vs. 54.72%, *P*=0.99). Unlike the results of phase III randomized controlled trials, Olanzapine, 10 mg, is more effective than aprepitant in the control of nausea during the DP [5]. Similarly, in one of the assessments and studies by Rumyantsev et al., the TC rate of 5 mg olanzapine administered consecutively for five days was also found to be superior to that of aprepitant during the OP (44.% vs. 24.0%, *P*=0.04) [17]. The reasons for these differences are complex, but they may be due to the overlap of the AP and DP of CINV induced by multiday administration of cisplatin, resulting in changes in the nausea pattern and increases in the TP rate after cisplatin chemotherapy, which makes it more difficult to observe the differences [7, 8]. In addition, the doses of 5-HT3RA, DXM, and olanzapine are different. The specific time, date, and days in this paper will also have a certain impact on the results. The TP rate in DP (54.81%) was significantly lower than that in AP (94.23%), suggesting that the prevention of nausea during DP was more difficult and, thus, is worthy of a further clinical study. In addition, the CR rate during the OP after research and discussion shows that this result is much better than the previous research results [5]. Here, the AP was defined as 24 hours, the time cut-off points between AP and DP in multiple-day cisplatin-induced chemotherapy are different, which may also directly affect the CR rate. Gao found that when the cut-off point of the AP was changed from 24 hours to 72 hours, the CR decreased by about 20% [2, 18]. On the other hand, since younger age and female gender have both been found to be high-risk factors for CINV [19], the relatively high proportion of male and elderly patients in this study may have led to a higher CR rate.

This study confirmed that olanzapine 5 mg is as effective as olanzapine 10 mg in preventing CINV, as found in the previous phase II study conducted by Takako et al. [6]. In another randomized double-blind comparative study, 10 mg (10 mg olanzapine, ondansetron, and dexamethasone) or 5 mg olanzapine (5 mg olanzapine, ondansetron, and dexamethasone) group and the aprepitant (aprepitant, ondansetron, and dexamethasone) group were 65.2% vs. 66% (*P*=0.94) and 68% vs. 66% (*P*=0.83), respectively [20], which also supports the findings of this study, that 5 mg olanzapine is comparable to aprepitant in the prevention of CINV.

In terms of side effects, the incidence of drowsiness among the studies (88.46%) is higher than that in the study conducted by Hashimoto et al. and Suthinee et al., which may be due to different criteria for the evaluation of drowsiness [17, 21]. The relatively higher proportion of elderly patients in our study may lead to a higher incidence of drowsiness because the previous study has shown that olanzapine has a more obvious effect in elderly patients [22].

## 5. Conclusion

In summary, the efficacy of 5 mg olanzapine is comparable to aprepitant and has the advantage of an economic potency ratio in preventing CINV induced by multiple-day cisplatin chemotherapy. Although low-dose olanzapine was 97% cheaper to prevent CINV than aprepitant but may cause weight gain and new-onset diabetes mellitus with sedation [21, 22]. In future, it deserves further clinical study for using olanzapine to improve nausea control during the DP and also pay attention to olanzapine-related side effect management.

## Figures and Tables

**Table 1:** Antiemetic administrations.

	Day 1	Day 2	Day 3	Day 4
Olanzapine antiemetic regimen	Olanzapine 5 mg po	Olanzapine 5 mg po	Olanzapine 5 mg po	Olanzapine 5 mg po
	Tropisetron 5 mg iv	Tropisetron 5 mg iv	Tropisetron 5 mg iv	
	DXM 10 mg iv	DXM 10 mg iv	DXM 10 mg iv	
Aprepitant antiemetic regimen	Aprepitant 125 mg po	Aprepitant 80 mg po	Aprepitant 80 mg po	
	Tropisetron 5 mg iv	Tropisetron 5 mg iv	Tropisetron 5 mg iv	
	DXM 5 mg iv	DXM 5 mg iv	DXM 5 mg iv	

**Table 2:** Baseline characteristics of patients in two groups (*n* (%)).

Characteristics	Olanzapine group (*n* = 104)	Aprepitant group (*n* = 106)	*P*
Age (years)	59.26 ± 8.865	60.01 ± 10.358	0.574
≥55	79 (75.96)	78 (73.58)	0.692

Gender			0.755
Male	64 (61.54)	63 (59.43)	
Female	40(38.46)	43(40.57)	

Smoking index			0.474
No smoking	39 (37.50)	47 (44.34)	
0∼400	13 (12.50)	9 (8.49)	
≥400	52 (50.00)	50 (47.17)	

Alcohol use			0.725
No consumption	48 (46.15)	50 (47.17)	
<4 drinks per week	38 (36.54)	34 (32.08)	
≥4drinks per week	18 (17.31)	22 (20.75)	

History of female pregnancy vomiting	13 (32.5)	11 (25.58)	0.487

History of motion sickness	17 (16.35)	21 (19.81)	0.514

Chemotherapy cycle			0.232
First cycle	27 (25.96)	37 (34.90)	
Second cycle	30 (28.845)	23 (21.70)	
Third cycle	17 (16.35)	23 (21.70)	
Fourth cycle	30 (28.845)	23 (21.70)	

Type of malignancies			0.755
Lung cancer	40 (38.46)	43 (40.57)	
Others	64 (61.54)	63 (59.43)	

**Table 3:** Comparison of FLIE index.

Items	Olanzapine regimen	Aprepitant regimen	*P*
Nausea FLIE score	51.75 ± 11.91	51.42 ± 11.59	0.837
Vomiting FLIE score	57.18 ± 11.37	57.33 ± 11.87	0.924
FLIE score	108.83 ± 21.65	108.69 ± 21.90	0.960

## Data Availability

No data were used to support this study.
